# Lipid Nanovesicle Platforms for Hepatocellular Carcinoma Precision Medicine Therapeutics: Progress and Perspectives

**DOI:** 10.1080/15476278.2024.2313696

**Published:** 2024-02-15

**Authors:** Brandon M. Lehrich, Evan R. Delgado

**Affiliations:** a Division of Experimental Pathology, Department of Pathology, University of Pittsburgh, Pittsburgh, Pennsylvania, USA; b Medical Scientist Training Program, University of Pittsburgh, Pittsburgh, Pennsylvania, USA

**Keywords:** Cell therapy, exosomes, extracellular vesicles, hepatocellular carcinoma, lipid nanovesicles, precision medicine

## Abstract

Hepatocellular carcinoma (HCC) is one of the leading causes of cancer-related mortality globally. HCC is highly heterogenous with diverse etiologies leading to different driver mutations potentiating unique tumor immune microenvironments. Current therapeutic options, including immune checkpoint inhibitors and combinations, have achieved limited objective response rates for the majority of patients. Thus, a precision medicine approach is needed to tailor specific treatment options for molecular subsets of HCC patients. Lipid nanovesicle platforms, either liposome- (synthetic) or extracellular vesicle (natural)-derived present are improved drug delivery vehicles which may be modified to contain specific cargos for targeting specific tumor sites, with a natural affinity for liver with limited toxicity. This mini-review provides updates on the applications of novel lipid nanovesicle-based therapeutics for HCC precision medicine and the challenges associated with translating this therapeutic subclass from preclinical models to the clinic.

## Introduction

Hepatocellular Carcinoma (HCC) is a growing global public health burden. HCC is the sixth most common cancer globally, with > 900,000 cases each year, and the third highest in cancer-related mortality, with > 800,000 deaths each year.^1^ HCC typically follows a sequalae of chronic liver disease, with the main etiologies including Hepatitis B and C virus (HBV & HCV) infection, alcohol-related disease, steatotic liver diseases (SLD) (e.g., metabolic dysfunction associated SLD [MASLD], diabetes mellitus, obesity), and toxin exposure (e.g., cigarette smoke, aflatoxin, liver fluke).^2^ HCC has a dismal prognosis with a 5-year overall survival (OS) rate of ~ 15–20% and <18 months median survival with current therapeutic paradigms.^3^ Very few patients are diagnosed at early stages where surgical resection/transplantation is feasible and nearly curative.^4^ In fact, the vast majority of patients are diagnosed with advanced disease, limiting their options to systemic agents, including tyrosine kinase inhibitors (TKIs) and, more recently, immune checkpoint inhibitors (ICIs). Despite ICIs demonstrating improved OS of roughly six months over TKIs, such as Sorafenib, the benefit is still marginal with only 25–30% response rates in patients.^5–7^

Therefore, novel targeted therapies used in conjunction with immunotherapy, in a precision-medicine based approach, may overcome HCC therapeutic resistance to ICIs in molecular subsets of patients. Lipid nanovesicle platforms, either liposome- (synthetic) or extracellular vesicle (natural)-derived, have demonstrated promise as drug delivery vehicles to the liver and for targeted cancer agents. These nanocarriers are ideal drug delivery vehicles which may be functionalized to harbor specific cargo molecules and “home” to specific tumor sites, with native affinity for liver with limited toxicity.^8,9^ This mini-review discusses HCC molecular subclasses and current treatment paradigms, along with applications of novel lipid nanovesicle-based therapeutics for HCC precision medicine with a focus on naturally-derived nanovesicle formulations, and the challenges associated with translating this new therapeutic subclass from preclinical models to the clinic.

## Genetic heterogeneity and immunologic landscape

Over the last decade, next-generation sequencing technologies have been utilized to profile genetic drivers of HCC, guiding the path toward precision medicine therapeutics. Our current understanding of the HCC genomic landscape includes major somatic mutations in TERT (~50%; telomere maintenance; promoter mutation and gain-of-function [GOF]), TP53 (~30%; cell cycle control; missense/nonsense; loss-of-function [LOF]), CTNNB1 (~30%; Wnt/β-catenin signaling; missense; GOF), ARID1A (~10%; chromatin remodeling; truncating/missense; LOF), and TSC2 (~10%; cell growth; deletions; LOF).^10,11^ Less common molecular drivers include FGF19 (~10%), AXIN1 (~6%), MYC (~6%), APC (~5%), and MET (~2%).^10,11^ Some of these mutations may not be mutually exclusive; however, mutations in Wnt/β-catenin pathway members and TP53 tend to be mutually exclusive events.^12^ This dichotomy also forms the foundation for defining the various molecular subclasses of HCC described here.

The two main molecular classification systems proposed are the G1-G6 system by Boyault et al.^13^ and the S1-S3 subgroups by Hoshida et al.^14^ Briefly, G1-G3 and S1-S2 subclasses represent proliferative/poorly differentiated tumors associated with chromosomal instability, high HBV viral load, and TP53 mutations, while G5-G6 and S3 subclasses represent non-proliferative/well-differentiated tumors associated with chromosomal stability, alcohol/HCV/NASH-driven HCC, and CTNNB1 mutations.^13,14^ More recently, HCC can be classified into inflamed (Hoshida S1-S2 subgroups) or non-inflamed (Hoshida S3) subgroups.^14,15^ The inflamed class of HCC (~25% of patients) demonstrates increased expression of gene signatures related to immune infiltration (i.e., cytotoxic T cells, tertiary lymphoid structures [TLS], IFN alpha and gamma signaling, and chemokines CXCL9, CXCL10), high immune checkpoint immunohistochemical expression, CTNNB1-mutated depleted, and enrichment of amplification in q13 locus (*CCND1, FGF19*).^15^ Additionally, the inflamed class can be further subdivided into either immune-active or immune-exhausted, with the immune-active subclass representing high adaptive immunity gene expression with improved survival and reduced rates of recurrence. The immune exhausted subclass demonstrates activated stroma and immunosuppressive gene set signatures.^16^ Overall, these classification systems illustrate how tumor genetics drive both tumoral heterogeneity and specific tumor microenvironments, which may be differentially susceptible to various systemic agents, and thus, may require tailored treatment options for patients informed by tissue and/or liquid biopsy.^17^

## Current treatment modalities and patient selection

For advanced HCC, current standard of care has shifted from the use of TKIs toward ICIs in the last decade. ICIs are monoclonal antibodies which block the interaction between immune checkpoint molecules (e.g., programmed death-ligand 1 [PDL1] on tumor cells interacting with programmed cell death protein 1 [PD1] on T cells) potentiating cytotoxic CD8+ T cell mediated tumor cell killing.^18^ The IMbrave 150 trial demonstrated 19.2 months median survival with atezolizumab (anti-PDL1 antibody) plus bevacizumab (anti-VEGF antibody) compared to 13.4 months median survival with sorafenib (TKI).^19^ Also, the HIMALAYA trial demonstrated 16.4 months median survival with the ICI combination of tremelimumab (anti-CTLA4 antibody) plus durvalumab (anti-PDL1 antibody) compared to 13.7 months median survival with sorafenib.^7^ Moreover, the CARES-310 trial demonstrated 22.1 months median survival with camrelizumab (anti-PD1 antibody) plus the VEGFR2-targeted TKI rivoceranib compared to 15.2 months median survival with sorafenib.^20^ However, despite the improved OS in ICI treated patients, response rates overall remain relatively low with only 25–30% of patients achieving objective response rates (ORR). Low ORRs are poorly understood but have been linked to patient tumor microenvironments with low tumor-infiltrating effector T lymphocyte density, high regulatory T cell density, and high expression of oncofetal genes.^21^ Thus, to improve these response rates, an individualized treatment approach is warranted to guide therapeutic selection based on underlying genetic alterations. This may be aided by tissue or liquid biopsy for key drivers of HCC tumorigenesis.^17^ However, an improved understanding of which genetic drivers influence the immune microenvironment resistant to ICI response is warranted for screening, along with needing an expanded arsenal of drugs targeting these underlying pathways to be used in conjunction with ICIs.

On a molecular basis, the Wnt/β-catenin pathway has been the most prominently studied pathway to evaluate ICI resistance, yet controversy remains whether all mutations in the pathway decrease immune infiltration to the same degree, and thus ICI resistance.^16, 21, 22^ Moreover, despite studies demonstrating the feasibility of prospective tissue genotyping to identify clinically actionable driver mutations, very few patients receive personalized therapeutic intervention.^10^ The major driver mutations in HCC are currently not actionable^23;^ therefore, efforts should be made to identify and stratify patients which may respond to current druggable targets, including FGF19/FGFR4, VEGF, TSC1/2, and MET inhibitors.^24,25^ Although none of these targets have shown clinical responses, these molecular events may be co-occurring in the background of strong drivers (e.g., TP53, CTNNB1), and thus a combination of therapeutics may need to be eventually employed. Thus, further studies are needed in clinically relevant animal models to determine the differential response of ICIs in combination with targeted therapy approaches in unique molecular subsets of HCC.

## Synthetic lipid nanovesicle drug delivery platforms for HCC

**Figure 1.**
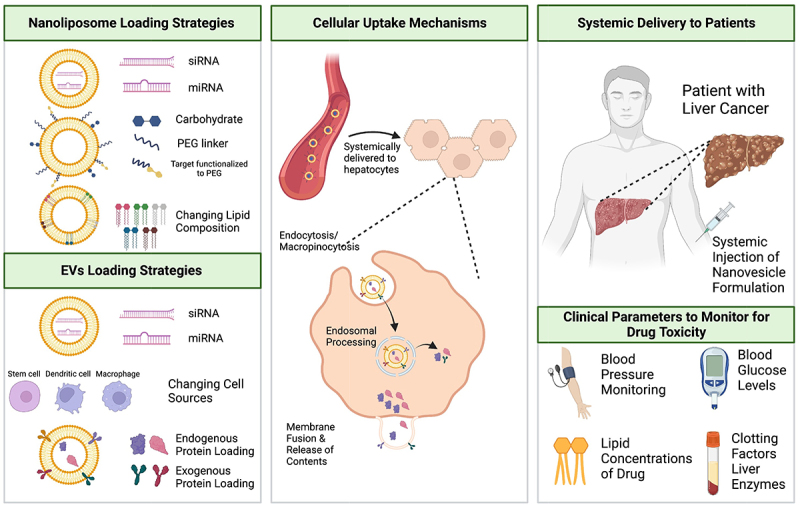
Schematic representation of nanoliposome and extracellular vesicle loading strategies, cellular uptake mechanisms of these drug delivery vehicles, and clinical parameters to monitor for toxicity in patients. Figure made in BioRender.

Synthetic lipid nanovesicles have conventionally been nanoliposome-based formulations containing distinct molecular entities, including either RNA interference (RNAi) technologies or chemotherapeutic drugs. Nanoliposomes typically size range between 10 nm to 200 nm in diameter and are composed of a phospholipid bilayer with or without cholesterol, resulting in an aqueous interior and an outer hydrophobic exterior.^26^ The main types of nanoliposomes include small unilamellar vesicles (<100 nm), large unilamellar (>100 nm), and multilamellar vesicles (>500 nm), with the former two more typically used for nanomedicine applications.^27^ Excellent reviews elsewhere discuss preparation methodologies (e.g., reverse-phase evaporation, freeze-thaw method, vaporization technique, and others) of nanoliposome formulations.^28,29^ Briefly, the phospholipid characteristics (e.g., degrees of unsaturation, quantity of fatty acid moieties, and others) and the number of cholesterol molecules can affect the membrane configuration.^30,31^ Further modifications to the nanoliposomal structure include the addition of either polyethylene glycol^32^ or surface ligands,^33^ which avoids host immune system elimination and improves cellular targeting, respectively. For cellular uptake, nanovesicles are internalized typically through endocytosis or phagocytosis, with nanovesicle structure influencing which mechanistic process.^34^ Efficient perfusion of the liver through its dual blood supply mediates optimal delivery, and lipid nanovesicle uptake is augmented due to its fenestrated endothelium. Additionally, opsonization by ApoE facilitates low-density lipoprotein (LDL) receptor (LDLR)-mediated uptake into hepatocytes (“endogenous targeting”), while engineering N-acetylgalactosamine (GalNAc)-PEG-lipid on the nanovesicle surface can target the asialoglycoprotein receptor (ASGPR) on hepatocytes (“exogenous targeting”), with both options providing efficient delivery to the liver.^35,36^ This well-characterized ApoE-LDLR endogenous hepatocyte targeting mechanism is the route by which Patisiran, the first FDA approved siRNA-based drug, facilitates its end-organ targeting to the liver and mechanism of action.^37^ The remainder of this section will discuss the applications of nanoliposomes as targeted drug delivery vehicles in various preclinical models of HCC as potential precision medicine therapeutic platforms (Figure 1).

The realization of using lipid nanovesicles as a targeted therapy delivery vehicle for liver cancer in humans was first achieved in 2013 by Tabernero and colleagues in their phase I study. This lipid nanovesicle (ALN-VSP) encapsulated siRNAs targeting vascular endothelial growth factor (VEGF) and kinesin spindle protein (KSP) to treat patients with liver metastases.^38^ Tumor regression was achieved in nearly 50% of the patients in the trial. These results, demonstrating the safety, tolerability, ability to achieve target downregulation in the liver, and short-term clinical responses underscore the importance and potential of using lipid nanovesicles for HCC therapy.

Nanoliposomes encapsulating RNA interference (RNAi) platforms, such as small interfering RNAs (siRNAs), microRNAs (miRNAs), or messenger RNAs (mRNAs) have been administered as drug delivery systems in preclinical models of HCC with considerable success in terms of safety, tolerability, and treatment response. Various groups have attempted to use RNAi to either target oncogenic genes involved in cell cycle regulation and cell proliferation/death pathways, or directly inhibit driver mutations deemed to be traditionally “undruggable.” For reviews on how RNAi platforms are processed following cellular uptake, we refer the interested reader to the following reviews.^39–42^ An example of directly targeting oncogenic factors is illustrated by work from Younis colleagues where they encapsulated both a siRNA to midkine (MK; a gene involved in many cellular pathways including apoptosis and angiogenesis^43^ and the chemotherapeutic, sorafenib, into a nanoliposome functionalized to contain 3 components: 1) SP94 peptide (specific to HCC cells), 2) YSK05 lipid (increased cytotoxic effects and limited endosomal escape), and 3) specific phosphatidylcholine/cholesterol ratio (improves liposome stability).^44^ They demonstrated both *in vitro* and *in vivo* that their nanoliposome had specific uptake to HCC cells over normal hepatocytes, potentiated sorafenib’s effects, and resulted in profound tumor regressions (~70%).^44,45^ Additionally, Woitok et al. delivered siRNA targeting Jun N-terminal kinase-2 (Jnk2), known to affect fibrosis progression, in lipid nanovesicle to mice with chronic liver disease and demonstrated decreased HCC premalignant nodules and a shift in the immune microenvironment of the diseased liver.^46^ Moreover, targeting key cellular pathways in HCC with siRNAs has also been feasible as demonstrated by the work from Fitamant and colleagues.^47^ They delivered nanovesicles containing siRNA to Yes-associated protein 1 (YAP), a key downstream transcriptional co-activator of Hippo signaling, resulting in tumor regression through directing hepatocyte differentiation to normal hepatocyte-like cells. Other groups have also delivered nanoliposomes containing siRNAs targeting PD-L1,^48^ T cell immunoglobulin mucin-3^49^ (Tim-3; immune checkpoint molecule), vascular endothelial growth factor^50^ (VEGF; angiogenic factor), alpha-fetoprotein^51^ (AFP; biomarker for HCC), cyclo-oxygenase-2^52^ (COX-2; important for prostaglandin synthesis in inflammatory processes), hypoxia inducible factor 1 subunit alpha^53^ (HIF1a), or RNA N^6−^methyladenosine (m^6^A) reader protein YTHDF1^54^ either alone or in combination with chemotherapeutics. Moreover, miRNAs can be packaged into nanoliposomes to target specific cellular pathways. For example, Zhao et al. loaded miR-375 and sorafenib in nanoliposomes to hinder autophagic processes and reduce tumor burden.^55^ Lastly, mRNAs may also be packaged into nanovesicles for HCC therapy. Lai et al. demonstrated that delivery of IL-12 mRNA in nanovesicles reduced tumor burden and prolonged survival of transgenic MYC-induced HCC mice.^56^ This effect was also associated with a shift toward a more anti-tumor immune microenvironment with increases in T helper cells and IFNγ expression.^56^ Similar effects were seen with mRNA for OX40L encapsulated nanovesicles.^57^ Overall, lipid nanoparticles provide an efficient platform to deliver both chemotherapeutics and gene therapy at subtoxic doses with high efficiency and stability.^44,53^

As previously discussed, modifying the outer shell of the nanoliposome can improve the delivery efficiency and targeting to the desired end organ. For targeting HCC cells specifically, various groups have functionalized nanoliposomes to target CXCR4 high expressing cells given its sorafenib resistance mechanisms. These studies have demonstrated reduced toxicity with targeted nanoparticles and synergistic effects when combined with chemotherapies, such as a sorafenib.^58–60^ Additionally, GalNAc-conjugated nanovesicles have demonstrated considerable success in highly relevant animal models of molecular subsets of HCC with the nanoliposomes encapsulating siRNAs to oncogenic drivers, such as CTNNB1.^61,62^ Also, the lipid configuration and inclusion of PEG/mannose into the membrane can also affect targeting to different liver cell types.^63^ Therefore, using targeting molecules on nanoliposome surface can improve the efficiency of tumor cell transfection and diminish off-target effects.

Moreover, another strategy is modifying the lipid composition of the liposome for controllable release of chemotherapeutic agents through stimuli responses. Examples of this include using either temperature sensitive,^64^ pH responsive,^65,66^ photo-sensitive, magnetic-sensitive, or ultrasound-guided lipids.^67^ In terms of temperature-sensitive lipids, Peng et al.^64^ utilized PF127 (copolymer) which has temperature-sensitive properties and aids in degrading the nanoliposome following photothermal conversion of IR-780 (a near-infrared [NIR] dye) also contained on the nanoliposome surface. This combination of PF127 and IR-780 allowed for efficient doxorubicin and sorafenib release at the tumor site *in vivo*. Also, as illustrated by Li et al.,^65^ interchanging the nanoliposome bilayer to include the cationic lipid (2E)-4-(dioleostearin)-amino-4-carbonyl-2-butenonic (DC), can allow for direct tumor cell internalization upon conformational change in the acidic tumor microenvironment, and subsequently release its cargo in the acidified endosome. This allowed for reduced drug toxicity and targeting of tumor cells over normal hepatocytes. Overall, the lipid composition can allow for improved pharmacokinetics and tumor cell internalization.

## Extracellular vesicle-based drug delivery platforms for HCC

Extracellular vesicles (EVs) are lipid nanovesicles (50 nm to >2000 nm) which are spontaneously produced by nearly all mammalian cells and released into extracellular fluid as part of autocrine, paracrine, and endocrine cell-to-cell signaling circuits.^68^ There are various EV subclasses, including exosomes (derived from endosomal membrane trafficking machinery), microvesicles (outward plasma membrane blebbings), and apoptotic bodies (from apoptotic processes). All EVs contain cargos comprising various membrane and soluble proteins, nucleic acid species, and metabolites, which are specific to their cell of origin. Once released into the extracellular milieu, EVs travel systemically until they make contact with and fuse with their target cell plasma membrane through various endocytic or phagocytic mechanisms.^69^ The natural ability for EVs to avoid immune system clearance, systemically travel to end organs, and package cargos within lipid bilayers has made them an attractive tool for drug delivery. Through the use of nanomedicine platforms, EV mimetics are being translated to the clinic as novel drug delivery vehicles. Various researchers have developed different EV mimetic technologies, either through modifying parental cells (e.g., stem cells, fibroblasts, immune cells) and isolating their EVs for delivery, or *ex vivo* loading of cargo components into EVs. This section will explore applications of EV mimetics for HCC precision medicine in preclinical models (Figure 1), and we refer to the reader to excellent reviews detailing techniques used for preparation of EV-based therapeutics, including their isolation and purification.^70–74^

The main class of EV mimetics utilized for HCC targeted therapy are siRNA- encapsulated EVs, which target specific mRNAs encoding oncogenic signaling proteins. Various groups have identified target genes, which when suppressed, may synergize with ICIs. One target is CD38, a transmembrane protein which is aberrantly expressed in many tumors and associated with a pro-inflammatory tumor microenvironment, and has been shown to be associated ICI response.^75,76^ EVs isolated from bone marrow mesenchymal stem cells packaged with siRNA to CD38 (via electroporation) reduced HCC tumor burden, metastatic potential, repolarized macrophages from M2 (immunosuppressive) to M1 (pro-inflammatory) phenotype, and improved ICI response.^75^ Other genes/pathways identified which have been targeted with siRNAs packaged in EVs, include components of the ferroptosis pathway (GPX4 and DHODH),^77^ cell cycle regulation (CDK1),^78^ JAK/STAT pathway (STAT6),^79^ and NFkB pathway (p50 subunit).^80^ Rather than directly targeting translation of molecules displayed on tumor cell surface mediating immunosuppression, another approach is targeting the underlying genetic mutation of the tumor cell. Matusda and colleagues designed an siRNA targeting CTNNB1 delivered within EVs.^81^ Using the Met/β-catenin mouse model (which represents ~ 10% of human HCC), they remarkably demonstrated that delivery of milk-derived EVs encapsulating siRNA to CTNNB1 (using transfection techniques) reduced tumor burden, in part through reversing the immunosuppressive tumor microenvironment driven by β-catenin, which allowed for synergy with ICIs. Another group utilized a similar platform, but functionalized the EVs to target EpCAM-positive HCC cells.^82^ These studies along with others previously mentioned^61, 62, 82^ provide direct evidence that therapeutically targeting oncogenic mutations with siRNAs are effective approaches to treat HCC. And, using EVs may have improved RNA delivery efficiency, unique targeting capabilities, and enhanced biocompatibility compared to synthetic nanovesicle platforms.^83–85^

Similar to siRNAs, miRNAs packaged into EVs offer another platform to target actively proliferating cancer cells. Many miRNAs have been implicated in HCC pathogenesis, including miR-21, miR-125b, miR-155, and miR-221/222.^86^ Particularly, miR-125b down-regulation is associated with worse overall survival.^87^ Baldari and colleagues isolated EVs (via polymer-based methods) from adipose-derived stromal cells (ADSCs) engineered to express miR-125b with a unique “ExoMotif” sequence that increases release of miR-125b into EVs.^88^ These EVs were delivered *in vitro* to HepG2 and HuH-7 cells and reduced cell proliferation, along with expression of p53 signaling pathway components.^88^ In another study, Mahati and colleagues loaded mesenchymal stem cell (MSC)-derived EVs with miR-26a (via electroporation) and observed impaired cell proliferation and migration *in vitro*, along with reduced tumor burden in subcutaneous HCC models.^89^ Lastly, Ellipilli and colleagues demonstrated that combined Paclitaxel and miR-122 (liver specific miRNA; reduced levels shown in HCC) administration within GalNAc-EVs reduced tumor burden in multiple mice xenograft HCC models.^90^ Complementary to RNAi, another strategy for EV therapeutics includes exogenous or endogenous small molecule and protein loading. Exogenous protein loading of EVs has been excellently reviewed elsewhere, but includes techniques such as mixing, sonication, electroporation, freeze-thaw cycles, and extrusion, with sonication and extrusion being the most efficient.^91–94^ Monoclonal antibodies, nanobodies, and various cytokines can even be packaged into EVs to target specific immune checkpoint molecules to induce native immune activity.^95,96^ However, these techniques may damage the membrane integrity of EVs.^92,97^ Endogenous protein loading into EVs is a novel technique which hijacks cell signaling cascades to load particular payloads into EVs, which can be isolated, and subsequently administered as therapeutics. Different groups have utilized the ability of FK506 binding protein (FKBP) and FKBP12–rapamycin-binding (FRB) domain to heterodimerize following rapamycin administration.^98,99^ The FRB domain is fused to the protein of interest via a GGSGG linker, and the FKBP domain is fused to a canonical EV protein (e.g., CD81 or CD63) via the N-myristoylation sequence to facilitate protein entry into EVs. Cell lines can be modified to express these fusion proteins and EVs can be isolated and delivered *in vivo* for effective protein delivery.^98,99^ Small molecule/chemotherapeutic agent packaging into EVs have demonstrated potential, including the use of doxorubicin,^100^ norcantharidin,^101^ and sorafenib.^102^ Additionally, Cas9 ribonucleoprotein can be packaged into EVs and delivered *in vivo* to liver, offering avenues for HCC gene therapy.^103–105^ Overall, these methods of protein/small molecule packaging are appealing options for therapeutic delivery to liver.

In the last two decades, recombinant Adeno-associated viruses (AAVs) have been explored as gene delivery vehicles for cancer due to their ability to target many cell types and long-lasting gene expression.^106^ More recently, EVs have been shown to be associate with isolated AAVs (termed “vexosomes”) during virus isolation from cell-culture media. These vexosomes have become an alternate gene delivery vehicle.^107,108^ Moreover, vexosomes protect AAVs from antibody neutralization, a major issue for AAV *in vivo* translation.^109^ Khan et al. isolated AAV6-derived vexosomes (via ultracentrifugation) containing an inducible caspase 9 (iCasp9), which upon delivery with a prodrug (AP20187), results in impaired HCC cell proliferation *in vitro* and tumor cell death *in vivo* via apoptosis.^110^ Overall, vexosomes are another gene therapy-based EV mimetic technology which are highly efficient delivery vehicles, require lower therapeutic doses than AAVs, and are not cumbersome to manufacture.

Lastly, EVs isolated from allogeneic or autologous cell sources are another therapeutic option for HCC. Kim and colleagues have demonstrated that EVs isolated from natural killer (NK) cells, which contain proteins important for mediating immunogenic cell death, can functionally impair HCC growth *in vitro* and *in vivo*.^111^ These NK-EVs (isolated via ultracentrifugation) express granzyme B, FasL, and TRAIL and mediate apoptosis through inducing caspase-3, 7, 8, and 9 upon internalization in tumor cells. Additionally, another cell type with promise as a therapeutic source of EVs are ADSCs. Wu and colleagues revealed that ADSC-EVs (isolated via ultracentrifugation of culture media) decreased hepatic fibrosis and glutamine synthetase levels, suggesting that this may have therapeutic potential in subsets of HCC.^112^ Moreover, another cell type which has demonstrated promise are dendritic cell (DC)-derived EVs. The pathogenesis of CTNNB1-mutated HCC involves defective recruitment of DCs,^113^ likely making DC-EVs an interesting platform as an HCC therapeutic. Lu and colleagues systemically administered DC-EVs in three different HCC models and observed shifts in the tumor microenvironment such as increases in cytotoxic CD8 T-cells and fewer immunosuppressive T regulatory cells, which associated with tumor regression.^114^ Lastly, M1 macrophages-derived EVs loaded with docosahexaenoic acid have been shown to induce ferroptosis and reduce tumor burden in orthotopic HCC models.^115^ Therefore, EVs isolated from allogeneic sources have intrinsic capabilities to alter tumor cell survival and growth. However, autologous-derived EVs may have improved tumor targeting properties. Villa et al. illustrated that EVs derived from blood plasma of cancer patients had selective uptake into associated patient-derived xenograft (PDX) mouse models.^116^ Therefore, autologous EV sources may be another pipeline for manufacture with improved tumor-specific targeting properties.

## Challenges in good manufacturing practices for nanovesicle therapeutics

Many of the challenges of translating nanovesicle therapeutics are shared between synthetic and natural platforms; however, this section will focus on the nuances associated with translating EV-based therapeutics. The first consideration is isolation purity. Current clinical Good Manufacturing Processes (GMP) of therapeutic EVs may lead to downstream isolation of contaminants (e.g., viral) from cell culture supernatants.^117^ For regulatory agency approval of EVs, a complete biochemical characterization is required for biologics, which remains incomplete due to technological limitations and EV isolation best practices.^117^ Additionally, given EVs are a cell-free therapy, the mechanisms of cellular uptake/targeting, cargo delivery/release, and an understanding of the precise bioactive and nonactive components are unclear.^117,118^ Whether the membrane lipids/proteins, or the proteins/nucleic acids in the lumen, or both, contribute to the intended therapeutic effect is not determined. Therefore, extensive functional assays, “–omic,” and imaging platforms are needed to fully elucidate and differentiate the physiochemical properties and bioactivity of EVs. The International Society for Extracellular Vesicles (ISEV) has established guidelines for clinical GMP of therapeutic EVs.^119^

The second consideration is cellular source and cell culture ecosystems of therapeutic EVs. As discussed in the previous section, cellular sources of therapeutic EVs for cancer can include either stem cells, immune cells, or nonparenchymal/stromal cells. Each of these cell types require different culture methods and release differing quantities of EVs. Additionally, cell culture practices of these cell types typically include utilizing fetal bovine serum (FBS) as a culture media supplement, which presents challenges due to introducing FBS-derived EVs into the pool of cell culture-derived EVs.^120^ Simply, this contamination means that upon isolation of EVs from the cell culture supernatant, the final EV fraction will contain both EVs from the FBS and the cultured cells.^120^ To circumvent these issues, the use of EV-depleted FBS or serum-free culture conditions have been proposed, with each providing their own inherent limitations, including cell death, incomplete elimination of FBS-derived EVs, and changes to cellular differentiation/state.^120,121^ Moreover, when culturing cells, the passage number, cell seeding density, and timing of media harvest can contribute to heterogeneity in cultured cells, and thus EVs isolated.^117,122^

The third consideration is the scale of manufacturing. For mass production of EVs, unique culture systems are needed, such as stacked culture vessels or bioreactors.^117,118^ Also, each EV isolation protocol (e.g., ultracentrifugation, precipitation, size-exclusion chromatography, and filtration) present differences in efficiency, quantity, purity, and quality of final EV formulations.^123^ For example, although centrifugation-based approaches improve EV purity, this is at the expense of cost and time.^120^ Lastly, with high-volume manufacturing, evaluating differences in batches is also important to consider.^117^

## Oncology clinical trials implementing nanovesicle platforms

The translation of lipid nanoparticles and EVs to clinical practice as HCC therapies has not moved swiftly. Currently, EVs are being studied as diagnostic biomarkers^124^ for HCC to detect initial diagnosis, response to therapy, and disease recurrence^125^ using DNA mutations^126, 127^ or methylation^128^ patterns, mRNA^129^/miRNA signatures,^130^ or proteins^131, 132^ encapsulated in their lumen. This section will briefly cover in-human studies in oncology which has successfully translated nanovesicle therapeutic platforms to the clinic. To investigate whether lipid nanovesicles were actively being translated into clinical trials, we surveyed the clinicaltrials.gov website to search for active or terminated trials. A review of the clinicaltrials.gov website for clinical trials related to “cancer” and “exosomes” yielded 132 studies, with 7 unique studies focusing on therapeutic applications (Table 1). Additionally, a review for clinical trials related to “cancer” and “nanovesicle” yielded 12 studies, with 7 unique studies focusing on therapeutic applications (Table 1). Overall, there are few trials investigating the therapeutic potential of lipid nanovesicle platforms in HCC space. Notably, Omega Therapeutics is leading their phase I/II MYCHELANGELO™ trial (NCT05497453) evaluating OTX-2002 as monotherapy or in combination with HCC standard of care (TKIs or ICIs), which is an mRNA therapeutic encapsulated in lipid nanovesicle which decreases c-MYC gene expression through modifying the c-Myc transcript via epigenetic modulation.^133^ They most recently (September 2023) have described preliminary results in 8 patients and observed on-target effects with associated decreases in c-MYC gene expression.^134^ This signals the transition of siRNA/mRNA lipid nanovesicle therapeutics from the preclinical to clinical realm to target traditionally “undruggable” oncogenic drivers to be used in conjunction with standard of care agents (i.e., TKIs or ICIs).

**Table 1.** Clinical trials registered on clinicaltrials.Gov website for use of lipid nanovesicles and extracellular vesicles in oncology.

Name	Identifier	Stage	Location	Clinical Setting	Agent(s) Utilized	Active or Completed
Lipid Nanovesicle Based Therapeutics						
A Phase I First in Human Study to Evaluate the Safety, Tolerability, and Pharmacokinetics of WGI-0301 in Patients With Advanced Solid Tumors	NCT05267899	Phase I	Valkyrie Clinical Trials (Los Angeles) Innovative Clinical Research Institute (Whittier, CA)	Any solid tumor	WGI-0301 is a lipid nanoparticle containing Akt-1 antisense oligonucleotide	Active
Dose Escalation and Efficacy Study of mRNA-2416 for Intratumoral Injection Alone and in Combination With Durvalumab for Participants With Advanced Malignancies	NCT03323398	Phase I	Multi-site ModernaTx	Relapsed/Refractory Solid Tumors or Lymphoma	mRNA-2416 is a lipid nanoparticle containing mRNA encoding for OX40L	Terminated
TKM 080301 for Primary or Secondary Liver Cancer	NCT01437007	Phase I	National Institutes of Health Clinical Center	Primary liver cancer of liver metastases	TKM-080301 is a lipid nanoparticle containing siRNA against PLK1 (polo-like kinase-1)	Completed
Dose Escalation Study of mRNA-2752 for Intratumoral Injection to Participants in Advanced Malignancies	NCT03739931	Phase I	Multi-site ModernaTx	Relapsed/Refractory Solid Tumors or Lymphoma	mRNA-2752 is a lipid nanoparticle containing mRNA encoding for OX40L, IL-23, and IL-36 g	Active, Recruiting
Phase I, Multicenter, Dose Escalation Study of DCR-MYC in Patients With Solid Tumors, Multiple Myeloma, or Lymphoma	NCT02110563	Phase I	Multi-site Dicerna Pharmaceuticals	Solid Tumors Multiple Myeloma Non-Hodgkins Lymphoma Pancreatic Neuroendocrine Tumors PNET NHL	DCR-MYC is a lipid nanoparticle containing siRNA to MYC oncogene	Terminated
First-in-Human Study of INT-1B3 in Patients With Advanced Solid Tumors	NCT04675996	Phase I	Multi-site InteRNA	Solid Tumor	INT-1B3 is a lipid nanoparticle containing miRNA-193a-3p	Active, Recruiting
A Phase 1/2 Study to Evaluate OTX-2002 in Patients With Hepatocellular Carcinoma and Other Solid Tumor Types Known for Association With the MYC Oncogene (MYCHELANGELO I)	NCT05497453	Phase I/II	Multi-site Omega Therapeutics	HCC	OTX-2002 is a mRNA therapeutic called an Omega epigenomic controller which modulates MYC gene expression; tested as monotherapy and in combination with standard of care	Active, Recruiting
Extracellular Vesicle Based Therapeutics						
Study Investigating the Ability of Plant Exosomes to Deliver Curcumin to Normal and Colon Cancer Tissue	NCT01294072	Phase I	University of Louisville Hospital	Colon Cancer	Curcumin alone in capsule form (Arm 1), Curcumin combined with plant exosomes (Arm 2), or No intervention (Arm 3)	Active, Recruiting
Trial of a Vaccination With Tumor Antigen-loaded Dendritic Cell-derived Exosomes (CSET 1437)	NCT01159288	Phase II	Gustave Roussy, Cancer Campus, Grand Paris	Lung Cancer	Vaccine with tumor antigen-loaded exosomes derived from dendritic cells	Completed
Edible Plant Exosome Ability to Prevent Oral Mucositis Associated With Chemoradiation Treatment of Head and Neck Cancer	NCT01668849	Phase I	James Graham Brown Cancer Center, University of Louisville	Head and Neck Cancer	Plant (grape) exosomes to prevent oral mucositis typically observed following chemoradiation	Completed
An Open, Dose-escalation Clinical Study of Chimeric Exosomal Tumor Vaccines for Recurrent or Metastatic Bladder Cancer	NCT05559177	Phase I	Fudan University Pudong Medical Center	Bladder Cancer	Chimeric exosomal vaccines prepared from autologous sources from differentiated blood monocytes to antigen presenting cells	Active, Recruiting
A Study of exoASO-STAT6 (CDK-004) in Patients With Advanced Hepatocellular Carcinoma (HCC) and Patients With Liver Metastases From Either Primary Gastric Cancer or Colorectal Cancer (CRC)	NCT05375604	Phase I	City of Hope National Medical Center Memorial Sloan Kettering Cancer Center Sarah Cannon Research Institute Codiak Biosciences	Hepatocellular carcinoma and liver metastases	CDK-004 is a STAT6 antisense oligonucleotide in cell-derived exosomes	Active, not recruiting
Antisense102: Pilot Immunotherapy for Newly Diagnosed Malignant Glioma	NCT02507583	Phase I	Thomas Jefferson University Hospital	Glioma	IGF-1 R/AS ODN is an Insulin-like growth factor receptor-1 antisense oligonucleotide in exosomes derived from malignant glioma cells	Completed
iExosomes in Treating Participants With Metastatic Pancreas Cancer With KrasG12D Mutation	NCT03608631	Phase I	MD Anderson Cancer Center	Metastatic Pancreatic Cancer	Exosomes derived from mesenchymal stromal cells with siRNA to KrasG12D mutation	Active, Recruiting

## Conclusions and future perspectives

Lipid nanovesicles are next-generation drug delivery vehicles swiftly becoming part of the oncologist armamentarium. Compared to the administration of “naked” drug, encapsulated drug within lipid nanovesicles allows for reduced toxicity, improved biocompatibility, and improved *in vivo* efficacy through enhanced delivery to end-organ and target cell internalization. Several studies have illuminated the potential of lipid nanovesicles, both synthetic and natural, as drug delivery platforms in preclinical models and in patients, with several companies licensing these technologies from academia and translating their products to the clinic. These platforms are ideal drug delivery vehicles for treating various liver pathologies, including cancer, due to the liver’s inherent dual blood supply and fenestrated endothelium to allow for efficient systemic administration and hepatocyte delivery, respectively. Also, these nanovesicles are opsonized by ApoE and recognized by the hepatocyte LDLR for efficient targeting. Or functionalization of the nanovesicle may allow for directed cell-type specificity.^135^

There are distinct advantages and disadvantages of each platform (Table 2). To improve the translation of this new EV class of biologics to the clinic, there are several technical challenges, including improving isolation techniques, component characterization, and manufacturing.^70,117^ Additionally, an enhanced understanding of the factors lending toward high biocompatibility of EVs may augment the development and translation of synthetic nanovesicles.^117^ Despite these challenges, the future is bright for nanovesicle therapeutic applications in oncology, particularly EVs, and as technology advances, these roadblocks will only become surpassed and push these biologics toward clinical practice. For translation of EV therapeutics, lessons may be learned from some of the hurdles overcome by those involved in translating nanoliposome formulations.^136^ For example, for nanoliposomes, great detail was undertaken to understand how the composition of ionizable lipids, various active drug loading techniques, and the cholesterol composition in the membrane affected drug stability, and thus enhanced *in vivo* activity.^137^ Additionally, the size of the nanovesicle plays an important role in the ability to target the liver (and specific cell-type), with studies concluding <100 nm is ideal for hepatocyte delivery.^136,138^ Interrogation of all these different tunable characteristics of nanovesicles for EV-based drug delivery vehicles will ultimately improve their translatability to the clinic.

**Table 2.** Advantages and disadvantages of different nanovesicle platforms for liver cancer.

Advantages	Disadvantages
**Nanoliposomes**	
Endogenous targeting to liver via ApoE-LDLR uptake mechanism	May have premature clearance by immune system before reaching end-organ
Exogenous targeting to liver via GalNAc (and others) functionalization	Cell-type specificity is challenged by vesicle size and membrane receptor components
Can selectively encapsulate specific nucleic acid species of choice	Scale-up manufacturing may be issue with high-cost
Formulations already FDA approved for various liver pathologies	Long term durability and bioactivity of the encapsulated payload
**Extracellular Vesicles**	
Enhanced biocompatibility compared to nanoliposomes	May contain other bioactive components not otherwise appreciated contributing to therapeutic effect
Less off-target toxicity compared to nanoliposomes	Isolation techniques may result in impurities
May have improved cell-type targeting based on parental source of EVs derived	GMP standards not well established for industry mass production
Improved ability to evade host immune clearance compared to nanoliposomes	Lack of predictable and precise sizing may hamper translation as hepatocyte targeting needs <200 nm
